# Physiological Sensor Signals Classification for Healthcare Using Sensor Data Fusion and Case-Based Reasoning

**DOI:** 10.3390/s140711770

**Published:** 2014-07-03

**Authors:** Shahina Begum, Shaibal Barua, Mobyen Uddin Ahmed

**Affiliations:** School of Innovation, Design and Engineering, Mälardalen University, SE-72123 Västerås, Sweden; E-Mails: shaibal.barua@mdh.se (S.B.); mobyen.ahmed@mdh.se (M.U.A.)

**Keywords:** sensor fusion, case-based reasoning, Multivariate Multiscale Entropy, classification, mental state

## Abstract

Today, clinicians often do diagnosis and classification of diseases based on information collected from several physiological sensor signals. However, sensor signal could easily be vulnerable to uncertain noises or interferences and due to large individual variations sensitivity to different physiological sensors could also vary. Therefore, multiple sensor signal fusion is valuable to provide more robust and reliable decision. This paper demonstrates a physiological sensor signal classification approach using sensor signal fusion and case-based reasoning. The proposed approach has been evaluated to classify *Stressed* or *Relaxed* individuals using sensor data fusion. Physiological sensor signals *i.e.*, Heart Rate (HR), Finger Temperature (FT), Respiration Rate (RR), Carbon dioxide (CO_2_) and Oxygen Saturation (SpO_2_) are collected during the data collection phase. Here, sensor fusion has been done in two different ways: (i) decision-level fusion using features extracted through traditional approaches; and (ii) data-level fusion using features extracted by means of Multivariate Multiscale Entropy (MMSE). Case-Based Reasoning (CBR) is applied for the classification of the signals. The experimental result shows that the proposed system could classify *Stressed* or *Relaxed* individual 87.5% accurately compare to an expert in the domain. So, it shows promising result in the psychophysiological domain and could be possible to adapt this approach to other relevant healthcare systems.

## 1. Introduction

Biomedical signals if processed correctly and efficiently have potential to facilitate advanced monitoring, diagnosis and treatment planning. However, sensor signal could easily be contaminated with uncertain noises or interferences and responses to these signals could also be different for different persons. In such cases, when analyzing sensor measurements, multiple sensors could provide more robust and reliable decision. For instance, if one sensor measurement is influenced by a certain noise or interference another sensor could still support the system. Also, in manual analysis experts often relay on several sensor signals and apply fusion based on their experience to make their judgment.

In recent years, sensor data fusion is becoming an emerging technology and researchers are applying new methods and techniques to introduce sensor fusion in various domains. For example, Lee and Chung [1] have proposed a system for monitoring driver safety levels in smart phones based on data fusion. In the system, they have fused several parameters *i.e.*, heart rate variability, blood pressure, in-vehicle temperature, vehicle speed and percentage of eyelid closure. The authors have developed a Fuzzy Bayesian framework which continuously indicates the driver's capability level in real-time. Riera *et al.* [2] have presented a data fusion method for stress detection. A designed protocol has been used to induce stress to the subjects. Here, electroencephalography (EEG) and facial (corrugator and zygomatic) electromyography (EMG) signals have been recorded during the data collection phase. The authors have performed two analyses: for the preliminary analysis, Fisher Discriminant Analysis (FDA) has been applied to correlate the subjects' EEG features mainly alpha asymmetry and alpha/beta ratio with stress levels. In the second step, they have fused EEG and EMG data. The authors have chosen a fusion operator tree, which forms a tree structure in a recursive application. The experimental results show that using the statistical inference achieved classification accuracy is up to 79% whereas using the fusion based method classification accuracy is 92%. Deng Y. *et al.* [3] have discussed a combinatorial fusion method for feature selection in stress identification domain. According to the authors, various features can be obtained from sensors however, the challenge is to select features that are most relevant to diagnose stress. The proposed combinatorial fusion method fuses feature sets that are selected based on performance and diversity between two features. Later, the authors have compared the results with other methods such as linear discriminant function, support vector machine, k-nearest neighbour (kNN), naïve Bayes and C4.5. They have suggested that the fusion based method is an efficient approach and it improves performances of the stress identification system. A multi-sensor fusion framework using Kalman Filter for indoor-outdoor localization for limited resource mobile robots is presented in [4]. They have combined measurements from a global sensor and an internal measurement unit in an event-based scheduling using fewer resources such as execution time and bandwidth. The results from the experiment show that the performance of the proposed sensor fusion framework is similar to the methods using more complex extended kalman filter and unscented kalman filter. The authors also suggest that by the sensor fusion framework can be adapted for other platforms. Also, systems using traditional sensor fusion methods such as Kalman filter, voting technique, artificial neural network, clustering, and Bayesian inference are discussed in [5–11].

This paper presents a physiological sensor signal classification approach based on sensor fusion to determine mental state in terms of *Stressed* or *Relaxed*. Here, it investigates decision-level and data-level fusion in a Case-base classification system. In a Case-Based Reasoning (CBR) classification scheme five sensor measurements *i.e.*, Heart Rate (HR) from Electrocardiogram (ECG), Finger Temperature (FT), Respiration Rate (RR), Carbon dioxide (CO_2_) and Oxygen Saturation (SpO_2_) are combined at data-level fusion by applying the Multivariate Multiscale Entropy (MMSE) algorithm [12,13]. In the system, weighted average algorithm is applied for the decision-level fusion. In the experimental work, the main goal is to investigate whether the proposed approach, despite large individual variations, could classify *Stressed* and *Relaxed* individuals close to an expert.

The rest of the paper is organized as follows: Section 2 presents the materials and methods where the data collection and case-based reasoning are discussed. Section 3, describes the sensor signals classification approach using the decision-level fusion. Section 4, illustrates the sensor signals classification approach using the data-level fusion. Section 5, discusses the experimental results. Finally, Section 6 ends with concluding remarks.

## 2. Materials and Methods

To classify mental state in terms of Stressed or Relaxed the proposed approach applies case-based reasoning and to evaluate the system data are collected using a data collection procedure.

### 2.1. Data Collection

**Figure 1.**
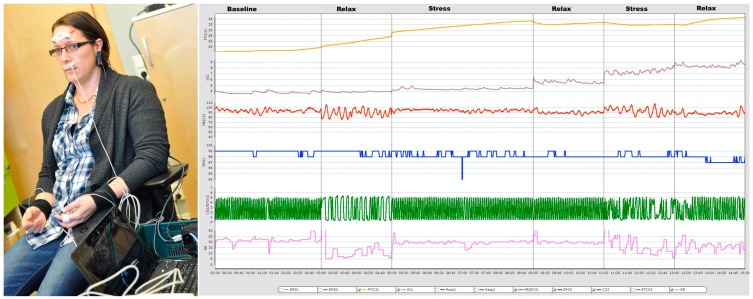
Experimental setup and five physiological signals during the physiological data profiling.

In this study, 16 measurements are collected from eight individuals (healthy and medication free), aged between 26 and 50 years. All participants were informed about the experimental setup (see Figure 1) before the data collection. Some contextual information such as age, hours sleeping over night before data collection, medication *etc.* have also been collected. Later, a medical expert uses these contextual data during stress classification. To collect data ***Airpass*** *and* ***C2*** devices have been used with another software ***“cStress”*** [14]. Five sensor signals *i.e.*, heart rate (HR), finger temperature (FT), respiration rate (RR), Carbon dioxide (CO_2_) and Oxygen Saturation (SpO_2_) are recorded, where ***Airpass*** is used for RR, CO_2_, and SpO_2_ signals and ***C2*** is used for HR, and FT. The data collection is carried out in two steps. In the first step, a psychophysiological stress profile (PSP) [15], which is a six-segments process, has been applied for profile data collection. Then, the second step of the data collection is performed in real road driving. In the real road driving, subjects are driving in a specific route from one start point to a specific destination and return to the starting point. However, to make the drivers stressed, a time constrain is imposed in each case to travel the predefined route and also they have been warned about the time remain to reach the destination. Figure 1 also displays a subject's five physiological signals during the profiling.

### 2.2. Methods

For the classification of the signals the approach works in two ways: (1) sensor signals classification using *decision-level* fusion; and (2) sensor signals classification using *data-level* fusion. In the *decision-level* fusion, features are extracted from each sensor signal separately through the traditional feature extraction approaches *i.e.*, considering time and frequency domains features. Therefore, in the CBR system five individual case-libraries have been constructed based on the five physiological signals. In the case retrieval step, it retrieves the most similar case from each case-library by matching each individual signal, that is, for example, a new FT signal matches with the previous solved FT signals and retrieves the top most case and uses it as a solution to the current case. Thus, the approach retrieves five classes for five signals and a weighted similarity function provides the final classification. In the *data-level* fusion, the signals are combined by means of a Multivariate Multiscale Entropy (MMSE) algorithm where the algorithm provides us with a number of features. The features that are extracted then form a new problem case and this new case is then feed into the CBR cycle. Here, the retrieval step retrieves the top most case and classifies the five-combined signal into *Stressed* or *Relaxed* class.

#### Case-Based Reasoning (CBR)

Learning from past experience and solve new problems by adapting similar previously solved cases is a cognitive model based on how humans often solve a large group of problems. A requirement is that the similarity of the case also indicates how easy the solution can be adapted to the current situation and reused.

**Figure 2.**
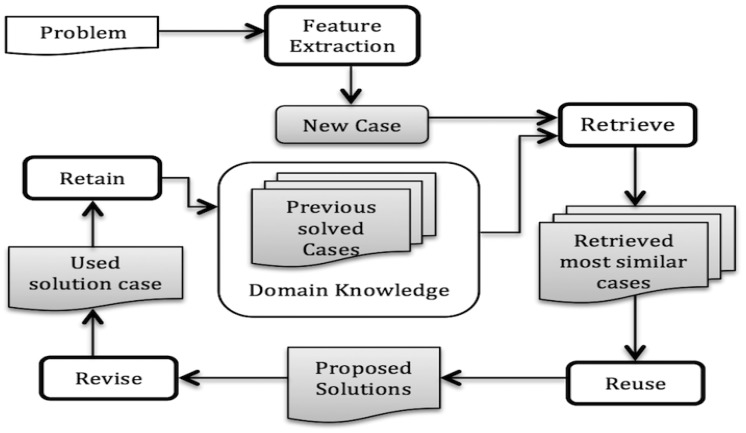
CBR cycle adapted from [16].

A case-based reasoning (CBR) [16,17] approach can work in a way close to human reasoning e.g., solves a new problem applying previous experiences, which is more common for doctors, clinicians or engineers. CBR has been applied successfully when the domain theory is not clear enough or even incomplete. It is getting increasing attention from the medical domain since it is a reasoning process that also is medically accepted [18–28]. For example, a clinician/doctor may start his/her practice with some initial experience (solved cases), then try to utilize this past experience to solve a new problem and simultaneously increases his/her case base. So, this method is getting increasing attention from the medical domain since it is a reasoning process that also is medically accepted. Aamodt and Plaza has introduced a life cycle of CBR [16] with four main steps as shown in Figure 2. Retrieve, Reuse, Revise and Retain present key tasks to implement such kind of cognitive model. In the retrieval step, for any new problem the system tries to retrieve the most similar case(s) by matching previous cases from a case base. If it finds any suitable case that is close to a current problem then the solution is reused (after some adaptation and revision if necessary).

In the CBR system, each case consists of two main parts: problem and solution. In this study, cases are constructed together with extracted features (as problem part) and classification classes (as solution part). Similarity of a feature values between two cases (*i.e.*, a target case and one case from library) is measured using the fuzzy similarity. In fuzzy similarity, a triangular membership function (*mf*) replaces a crisp value of the features for new and old cases with a membership grade of 1. In both the cases, the width of the membership function is fuzzified by 50% in each side. Fuzzy intersection is employed between the two fuzzy sets to get a new fuzzy set which represents the overlapping area between them:

(1) $$\text{sim}(C_{f},S_{f})=S_{f}\left(m1,m2\right)=\max\left(om/m1,om/m2\right)$$

The similarity between feature values of the old case (*S_f_*) and the new case (*C_f_*) is now calculated using Equation (1) where *m*1, *m*2 and *om* is the area of each fuzzy set [21]. The similarity between two cases is measured using the average of all the features that are to be considered. The function for calculating the similarity between two cases is presented in Equation (2):

(2) $$\text{sim}(C,S)=\sum_{f=1}^{n}w_{f}\times \text{sim}\left(C_{f},S_{f}\right)$$

where *C* is a current/target case, *S* is a stored case in the case base, *n* is the number of the attributes/features in each case, *f* is the index for an individual attribute/feature and sim (*C_f_, S_f_*) is the local similarity function for attribute *f* in cases *C* and *S*. It is noted that, in weight vector *w_f_* is also considered the weight of the two domains (*i.e.*, time and frequency features), which are defined by the domain expert and assumed to be a quantity reflecting importance of the corresponding feature.

## 3. Sensor Signals Classification Using Decision-Level Fusion

**Figure 3.**
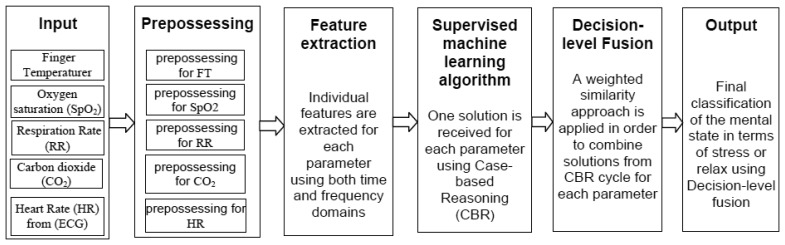
An overview of the classification scheme to identify mental state in terms of *Stressed* or *Relaxed* considering the decision-level fusion.

In the decision-level fusion, an overview of the classification approach is presented in Figure 3. Here, ECG, respiration rate, oxygen saturation and CO_2_ are taken from the ***Airpass*** sensor and finger temperature signal is collected from the ***C2*** sensor.

Before the feature extraction, each sensor signal has been pre-processed to handle artifacts or noises in the signal. Infinite impulse response (IIR) filter and smoothing running average method in the ***cStress*** software have been used for handling artifacts. Then, some erroneous values caused by artifacts have been replaced by the previous sample values. The signals are then used to extract time and frequency domain features. Section 3.1 presents the feature extraction procedure. The extracted features are then used to build a case library for the CBR classification system. For each signal, a solution is received considering the top most similar cases from each case-library. Finally, a weighted similarity function is used to combine the solutions and hence the system gets the final classification.

For the decision-level fusion, the system uses the weighted average similarity [29] algorithm. In this experiment, first a similarity value for a single signal source has been calculated using CBR. Then the fusion method is applied as a second level similarity calculation. Here, based on individual classification accuracy of the signals' (*i.e.*, HR, RR FT, CO_2_ and SpO_2_) weights in a range from 1 to 10 have been considered for each individual similarity values. The weighted average method can be expressed by Equation (3):

(3) $$\text{sim}S=\frac{w_{hr}*S_{hr}+w_{rr}*S_{rr}+w_{ft}*S_{ft}+w_{co2}*S_{co2}+w_{\text{spo}2}*S_{\text{spo}2}}{w_{hr}+w_{rr}+w_{ft}+w_{co2}+w_{SpO2}}$$

Here, *w* is the weight, multiplied by associated similarity value and sum of the multiplied similarity values are divided by sum of the weights. Then, the new similarity value is used in the CBR classification.

### Features Extraction through Traditional Approaches

Feature extraction and selection have been conducted using the traditional approaches discussed in [18,19,21,23,26,27]. Here, time and frequency domains features are extracted from the HR, RR, SpO_2_ and CO_2_ signals. In the *time domain*, statistical features namely *maximum* value, *minimum value, arithmetic mean* and *standard deviation* are calculated. To extract the *frequency domain* features, first the power spectral density has been calculated from the squared amplitude of the discrete fourier transformation value of the data using the fast Fourier transform (FFT) algorithm and then scale it to a sampling frequency range *i.e.*, 4 Hz in this case. To apply the FFT algorithm, zero padding of data has been done so that the number of data samples becomes power of two. Later, from the power spectral density *Low frequency power, High frequency power, Low and High frequency power ratio*, have been calculated [30]. The frequency between 0.04 Hz and 0.15 Hz is considered as *Low frequency* and frequency between 0.15 Hz and 0.4 Hz is considered as *High frequency* [30]. The power in High and Low frequency region is calculated by the numerical integration of power spectral density of the corresponding frequency range. For RR, mainly *arithmetic mean* and *standard deviation* have been extracted as features in the time domain. In the frequency domain, *dominant respiration frequency (DRF) i.e.*, maximum energy frequency which lies between the frequency range of 0.1 Hz and 1.5 Hz [31] has been selected as a feature. For the finger temperature (FT) sensor signal, a derivative of slope is used to extract the important features [18,19].

## 4. Sensor Signals Classification Using Data-Level Fusion

**Figure 4.**
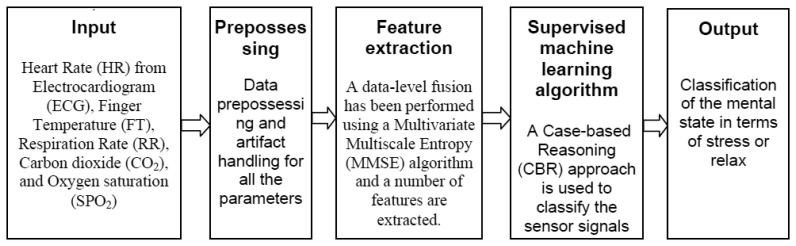
An overview of the classification scheme to identify mental state in terms of *Stressed* or *Relaxed* considering the data-level fusion.

In the data-level fusion, to extract features after pre-processing, all the five-sensor signals have been fused using the MMSE analysis algorithm. The measurement of the MMSE analysis has been applied as an input to the classification system. An overview of the classification scheme is presented in Figure 4.

One step of the MMSE algorithm is the coarse-grained process (average over increasing segment lengths) and it is measured up to scale factor 9 (see Section 4.1). As a result of the MMSE analysis, for each subject, we obtained a vector of nine elements. A case representation using MMSE analysis is shown in Table 1 where a new case is being matched with the top two most similar Cases *i.e.*, 4 and 14 from the case library by using the fuzzy similarity function. The bottom two cases, 13 and 15 are the least similar cases in the case library. Here, for each case, input features are obtained using the MMSE algorithm for the sensor signals (see Section 4.1) and an expert has classified each case as either *Relaxed* or *Stressed*.

### Features Extraction through MMSE

In this study, to extract feature five sensor signals namely HR, FT, RR, CO_2_ and SPO_2_ are fused using the MMSE analysis algorithm. The MMSE analysis algorithm works in two steps. The first step is to define temporal scales by averaging *p*-channel time series ${\left\{a_{k,i}\right\}}_{i=1}^{N},K=1,2,\ldots \ldots ,p$ using the coarse graining method (see Equation (4)). Here, *N* is the number of data points in each channel. In the second step, multivariate sample entropy, MSampEn has been evaluated for each coarse grained multivariate data. The algorithm constructs a composite delay vector from the coarse grained data to calculate MSampEn and its two important embedding parameters are m_k_ and τ_k_. A detail of the MMSE algorithm is available in [12,13].

In this study, for MMSE analysis, embedding vector parameters are considered as m_k_ = 2 and τ_k_ = 1 which are used in [3] for physiological signals. MMSE algorithm uses Equation (4) to obtain the coarse-grained process:

(4) $$b_{k,j}^{ɛ}=\frac{1}{ɛ}\sum_{i=\left(j-1\right)ɛ+1}^{jɛ}a_{k,i}\text{where}1\leq j\leq \frac{N}{ɛ}$$

where, *N* is number of data points in every channel, ${\left\{a_{k,i}\right\}}_{i=1}^{N},K=1,2,\ldots \ldots ,p$, is a *p*-varieties time series, **ε** is the scale factor, *k* = 1, …, *p* is the channel index and $b_{k,j}^{ɛ}$ is the coarse-grained data.

The MMSE analysis returns a linear vector based on the scale factor. To calculate MSampEn, for each *p* -variate time series a composite delay vector has been constructed using Equation (5):

(5) $$\begin{array}{c} a_{m}\left(i\right)=a_{1,i},a_{1,i+\tau_{1}},\ldots \ldots ,a_{1,i+\left(m_{1}-1\right)\tau_{1}},a_{2,i},a_{2,i+\tau_{2}},\\ \ldots \ldots ,a_{2,i+\left(m_{2}-1\right)\tau_{2}},a_{p,i},a_{p,i+\tau_{p}},\ldots \ldots ,a_{p,i+\left(m_{p}-1\right)\tau_{p}} \end{array}$$

where M = [m_1_, m_2_, m_3_, … , m_p_] ∈ R^p^ is the embedding vector, τ = [ τ _1,_ τ _2,_ …_,_ τ _p_] the time lag vector and composite delay vector *a_m_*(*i*) ∈ *R^m^* , where $m=\sum_{k=1}^{p}m_{k}$. Estimate of MSampEn is presented in Equation (6):

(6) $$\text{MSampEn}\left(M,\tau ,r,N\right)=-\ln\left[\frac{B^{m+1}\left(r\right)}{B^{m}\left(r\right)}\right]$$

where, *M* is embedding vector, τ is time lag vector, *r* is threshold and *N* is multivariate time series *B^m^* and *B^m^*^+1^ are the frequency of occurrence for the length m and *m* + 1 respectively.

**Figure 5.**
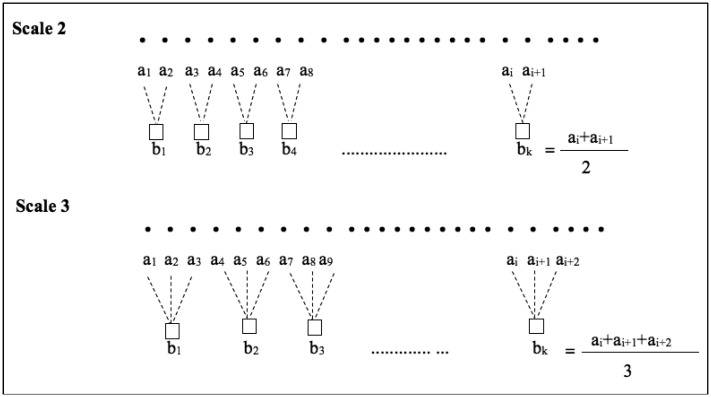
Illustration of the coarse-grained process in MMSE for scale factor 2 and scale factor 3.

The scale factor in coarse graining process is highly dependent on the length of data. However, the MMSE estimates are consistence for data length *N* ≥ 300 [12,13]. In this study, the scale factor has been chosen up to nine since at the scale factor 9 the shortest data series has greater than 300 data points. Figure 5 represents example of the coarse graining process in scale factor 2 and scale factor 3. Since we measure MMSE for scale factor up to 9 we obtained a vector of length 9 as a result of MMSE estimation for each recording. Thus, for each recording these nine values are considered as features.

## 5. Experimental Work

The evaluation process has been conducted in two ways: (i) CBR classification based on decision-level fusion using features extracted through traditional approaches; and (ii) CBR classification based on data-level fusion using features extracted through MMSE algorithm. Both the approaches have already been discussed in Section 5.1 and 5.2 respectively. In CBR, fuzzy similarity function is used for case matching [32], Leave-one-out approach *i.e.*, one case is taken out at a time to match against the remaining cases in the case library and kNN (K = 2) is applied to retrieve similar cases. For the evaluation, two top most similar retrieved cases are considered; if both the query and one of the two retrieved cases belonging to a similar class then the number of correctly classified cases is counted as 1. Here, it is indispensable to mention that each case have been classified by an expert as *Stressed* or *Relaxed*.

### 5.1. Sensor Signals Classification Using Decision-Level Fusion

The summary of the CBR weighted similarity classification considering the fuzzy similarity function is presented in Table 2. It can be seen from the table, when the top one case is matched *i.e.*, K = 1, the obtained accuracy for the CBR classification are: 75% for *Relaxed* and 50% for *Stressed* cases. On the other hand, when K = 2, the accuracy is achieved as 100% for the *Relaxed* cases and 75% for the *Stressed* cases. For all the classes (*i.e., Stressed* and *Relaxed*), achieved accuracy is 62.5% when K = 1 and 87.5% when K = 2. Table 3 shows weighted similarity value of the CBR classification in a confusion matrix. Table 4 presents the classification results for each individual signals.

### 5.2. Sensor Signals Classification Using Data-Level Fusion

In this step the experimental work has been carried out in two folds: (a) observation of the MMSE analysis; and (b) observation of the sensor fusion (MMSE) data using the Case-based classification.

#### 5.2.1. Observation of MMSE Analysis

In this experiment, five sensor signals (Heart rate, finger temperature, respiration rate, oxygen saturation (SpO_2_) and CO_2_) have been fused using the MMSE and entropy complexity has been computed up to scale 9. Then, an average entropy complexity value for each scale has been calculated both for the *Stressed* and *Relaxed* classes.

**Figure 6.**
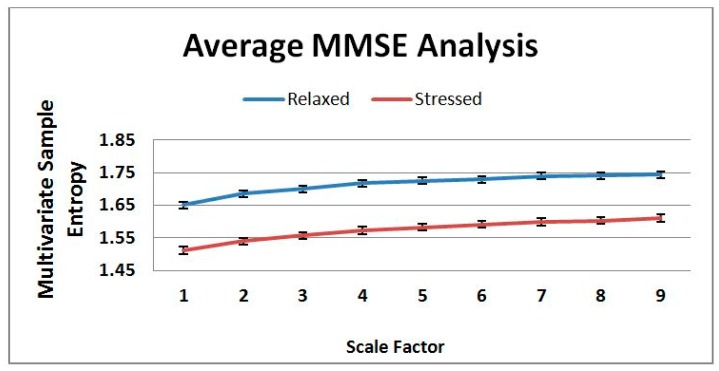
Average of the MMSE analysis with the standard deviation error bars for 8 *Relaxed* and 8 *Stressed* cases.

**Figure 7.**
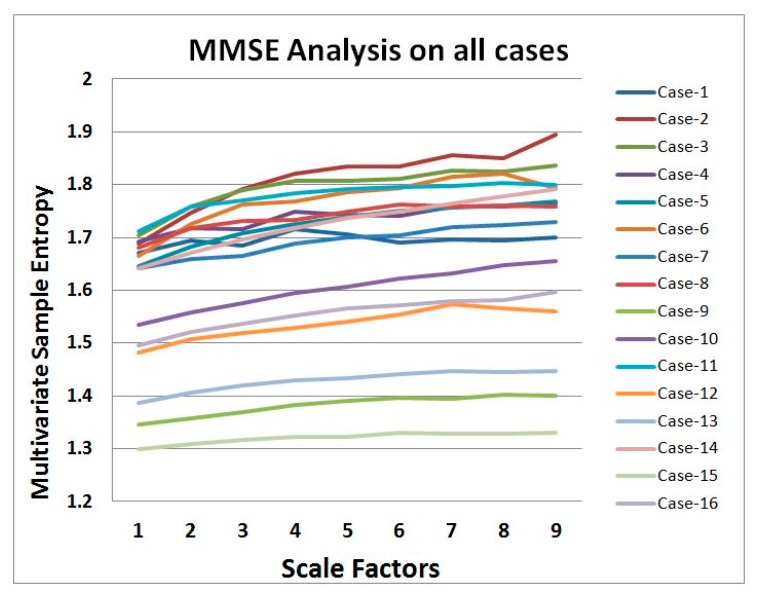
MMSE analysis for the 16 cases.

The average of the eight *Relaxed* and eight *Stressed* cases classified by an expert is shown in Figure 6. In the figure, it is clearly visible that the complexity value of the *Relaxed* cases are higher than the *Stressed* cases. Healthy systems have greater adaptability and functionality than disease systems. Different factors such as disease, aging, and drug toxicities degrade the physiologic information content and reduce adaptive capacity of an individual. The loss of complexity becomes a common feature in pathologic systems analysis. Consequently, Costa [33,34] has been established the hypothesis that healthy system has greater entropy complexity than disease system. Hence, the averages of the *Relaxed* and *Stressed* cases shown in Figure 6 have agreed with the hypothesis. Nevertheless, Figure 7 illustrates that the result of the MMSE analysis for the 16 cases vary a lot depending on the individuals. Since a large variation has been observed in Figure 7 this motivates to use a learning algorithm *i.e.*, CBR to classify the sensor data.

#### 5.2.2. Observation of the Sensor Fusion (MMSE) Data Using CBR

The summary of the sensor fusion based classification considering the fuzzy similarity function is presented in Table 5. It can be seen from the table that the results achieved are: 62.5% for the *Relaxed*, 37.5% for the *Stressed* cases when the top one case is matched (K = 1). On the other hand, when K = 2 the accuracy achieved are 100% for the *Relaxed* cases and 75% for the *Stressed* cases. In total, the accuracy achieved are: 50% and 87.5% considering K = 1 and K = 2 respectively. Considering, the fusion based classification a confusion matrix is presented in Table 6.

### 5.3. Comparison between the Data-Level and Decision-Level Fusion

A comparison between the data-level and decision level fusion using CBR has been accompanied considering the sensitivity and specificity analysis is shown in Table 7.

It can be seen from Table 7 using the *data-level* and *decision-level* fusion, out of 8 *Stressed* cases 6 are correctly classified. Sensitivity *i.e.*, percentage of cases that are identified as *Stressed*, is 75% and specificity *i.e.*, percentage of cases that are identified as *Relaxed*, is 100%. So, the overall obtained accuracy is 87% in both the cases *i.e., data-level* and *decision-level* fusion based classification.

## 6. Summary and Conclusions

This paper presents physiological sensor signal classification approach based on the *data-level* and *decision-level* fusion. It employs sensor fusion methods in a case-based classification system for classifying mental state in terms of *Relaxed* or *Stressed*. In reality, experts make judgment based on effectively fusing the information collected from different physiological sensor sources and they make assumptions and predictions based on their experiences or old cases. So, the main goal is to investigate whether in such classification systems is it possible to classify close to an expert's classification. In the proposed system, we have applied CBR since in CBR knowledge elicitation can be performed based on previous cases in a case library, especially suitable for domains where domain knowledge is not clear such as in classification of sensor signals. We have in the proposed system applied MMSE for the data-level fusion and weighted average algorithm for the decision-level fusion. However, the data-level fusion based on features extracted through MMSE algorithm is more autonomous than the decision-level fusion based on features extracted using traditional approaches. So, such decision-level fusion is encouraged to use while necessary expert knowledge is available. Several experimental works have been carried out to evaluate the system compare to an expert. In Figure 6, it shows that the MMSE algorithm can differentiate between stressed and healthy subjects. This supports the multiscale complexity loss theory with aging and disease or when a system is under constraints [35]. However, the MMSE analysis in Figure 7 illustrates that the individual variation is higher and underlying complexity analysis does not follow any rules which motivates to use a learning algorithm such as CBR in such systems. An evaluation of the CBR system based on individual sensor signal has also been performed in this study, see Table 4. It can be seen from the table, that the classification accuracy using HR, RR, FT, CO_2_ and SPO_2_ are 75%, 87.5%, 75%, 81.25% and 75% respectively. These results imply that all individual parameters except the RR parameter provide less accuracy than both using the decision-level and data-level fusion. Using the sensor fusion methods the proposed CBR system provides 87% classification accuracy compare to an expert. Moreover, sensor fusion provides us more reliable and information-rich judgment. Thus, the proposed fusion based approach could be of value to the systems where signals are coming from multiple sources.

## Figures and Tables

**Table 1.** An example of a case representation.

**Cases**	**Problem Description (Input Features after Applying MMSE Analysis)**									**Solution Description (Output class)**

	**F_1_**	**F_2_**	**F_3_**	**F_4_**	**F_5_**	**F_6_**	**F_7_**	**F_8_**	**F_9_**	
Case 4	1.6929	1.7180	1.7163	1.7482	1.7438	1.7416	1.7577	1.7583	1.7641	***Relaxed***
Case 14	1.6420	1.6712	1.6964	1.7172	1.7366	1.7491	1.7648	1.7779	1.7907	***Stressed***
Case 13	1.3867	1.4066	1.4203	1.295	1.4338	1.4421	1.4472	1.4452	1.4474	***Stressed***
Case 15	1.2992	1.3087	1.3161	1.3229	1.3233	1.3299	1.3284	1.3274	1.3296	***Stressed***

**Table 2.** Representation of percentage of correctly classified cases by the CBR weighted similarity.

**Case Classes**	**CBR (Weighted Similarity)**	
Criteria/Indices	K = 1	k = 2
8 Relaxed cases	75%	100%
8 Stressed cases	50%	75%
Total on 16 cases	62.5%	87.5%

**Table 3.** Confusion matrix based on CBR weighted similarity classification.

	***Stressed***	***Relaxed***
***Stressed***	6 (75%)	2 (25%)
***Relaxed***	0 (0%)	8 (100%)

**Table 4.** Classification accuracy considering CBR with single signal source. Here, HR = Heart Rate, FT = Finger Temperature, RR = Respiration Rate, CO_2_ = Carbon dioxide and SPO_2_ = Oxygen Saturation.

**Case Classes**	**CBR with HR**	**CBR with RR**	**CBR with FT**	**CBR with CO_2_**	**CBR with SPO_2_**
8 Relaxed cases	75%	87.5%	87.5%	100%	75%
8 Stressed cases	87.5%	87.5%	62.5%	62.5%	75%
Total 16 cases	75%	87.5%	75%	81.25%	75%

**Table 5.** Representation of Percentage of correctly classified cases by the fusion based classification considering the fuzzy similarity function.

**Case Classes**	**Fusion Based Classification**	
Criteria/Indices	K = 1	K = 2
8 Relaxed cases	62.5%	100%
8 Stressed cases	37.5%	75%
Total on 16 cases	50%	87.5%

**Table 6.** Confusion matrix based on the fusion based classification.

	***Stressed***	***Relaxed***
***Stressed***	6 (75%)	2 (25%)
***Relaxed***	0 (0%)	8 (100%)

**Table 7.** A comparison of the data-level and decision-level fusion based on statistical analysis of the classifications.

	**Using Data-Level Fusion**	**Using Decision-Level Fusion**
Criteria/Indices	K = 2	K = 2
Total cases	16	16
Cases belong to Stressed group (P)	8	8
Cases belong to Relaxed group (N)	8	8
True positive (TP):	6	6
False positive (FP):	0	0
True negative (TN):	8	8
False negative (FN):	2	2
Sensitivity = TP/(TP + FN)	≈0.75	≈0.75
Specificity = TN/(FP + TN)	≈1	≈1
Accuracy = (TP+TN)/(P + N)	≈0.87	≈0.87
